# Neutralization Capacity of Tissue Alterations Caused by the Venoms of the Most Dangerous Scorpions in North Africa Using a Selective Antivenom

**DOI:** 10.3390/toxins16010016

**Published:** 2023-12-27

**Authors:** Bouchra Darkaoui, Mohamed Aksim, Ayoub Aarab, Ayoub Lafnoune, Soukaina Khourcha, Rachida Cadi, Ouafaa Aniq Filali, Naoual Oukkache

**Affiliations:** 1 Laboratory of Venoms and Toxins, Pasteur Institute of Morocco, 1 Place Louis Pasteur, Casablanca 20250, Morocco; darkaoui.bouchra@hotmail.com (B.D.); ayoublafnoune@gmail.com (A.L.); khourcha.soukaina9@gmail.com (S.K.); 2 Laboratory of Molecular Genetics, Physiopathology and Biotechnology, Faculty of Sciences Ain Chock, Hassan II University of Casablanca, B.P 5366 Maarif, Casablanca 20000, Morocco; rachidacadi@gmail.com (R.C.); ouafa.aniqfilali@gmail.com (O.A.F.); 3 Laboratory of Anatomic Pathology, The Regional Hospital Centre Hassan II, Agadir 80000, Morocco; mohamedaksim@gmail.com; 4 Laboratory of Anatomical Pathology Marrakech, Agadir 80000, Morocco; ayoub-aarab@hotmail.fr

**Keywords:** venom, scorpion antivenom, toxicity, neutralization, tissular alterations, immunohistochemistry

## Abstract

In North Africa, scorpion stings pose an urgent public health problem, particularly for children with high morbidity and mortality rates. The main species implicated are the *Androctonus mauretanicus* (*Am*), *Androctonus australis hector* (*Aah*), and *Buthus occitanus* (*Bo*). Immunotherapy is the specific therapeutic approach aimed at directly neutralizing toxins, despite their severity and rapid diffusion. In the present study, we evaluate, histologically and immunohistologically, the neutralization potency of the selective antivenom produced against, among other species, the *Am*, *Aah*, and *Bo* at the level of the tissue alterations in Swiss mice, as experimental subjects. Firstly, the lethal doses 50 test was conducted to assess the venom’s toxic activity, and then the median effective dose of the antivenom was determined against each venom. The histological and immunohistological analyses were performed by injecting the sublethal dose of venom, the complex venom and antivenom, or the antivenom 2 h following inoculation of venom. Our study revealed the highest toxicity of the *Am*, followed by the *Aah* and then the *Bo* venom. The neutralizing ability and effectiveness of the antivenom to completely or partially neutralize the tissular damages were demonstrated in all organs studied: brain, heart, lungs, liver, and kidneys. Our results highlighted the important cytoplasmic and membranous staining in the heart compared to the brain tissue for the three scorpion venoms. Therefore, the scorpionic antivenoms are able to reach their target even at the tissue level. Immunotherapy represents the specific and recommended treatment against the scorpionic stings in North Africa.

## 1. Introduction

Scorpionism is a serious public health problem on a disturbing scale in the tropical and subtropical areas between latitudes 50° N and 50° S. North Africa is one of the areas that has recorded the most cases of envenomation. However, the incidence of scorpion envenomation is estimated at 1,500,000 cases worldwide annually, with 2600 deaths, and is mainly limited to 4 highly endemic regions: Mexico, South America to the East of the Andes, North Africa, and the Near and Middle East [1,2]. Morocco is among the main North African countries affected, besides Algeria and Tunisia. The prevalence of scorpionism is correlated with the geographic distributions of approximately 2621 species of scorpions that have been identified worldwide, belonging to 23 families [3]. Almost all dangerous species belong to the *Buthidae* family (88%), including the three species in North Africa: *Androctonus mauretanicus* (*Am*), known for the high toxicity of their venom and representing the endemic species in Morocco. The *Am* is responsible for the greatest number of fatal envenomations, especially in children. *Androctonus australis hector* (*Aah*) is considered one of the most dangerous scorpions in North Africa, with its distribution area in Algeria, Libya, and Tunisia. *Buthus occitanus* (*Bo*) is among the species with a very wide distribution in North Africa, particularly in Morocco, Algeria, and Tunisia [4,5,6,7,8]. The *Am* and *Bo* scorpions are the most dangerous species in Morocco [6,9].

Scorpion venom is a complex mixture of compounds with various biological activities, including enzymes, mucopolysaccharides, nucleotides, amino acids, and others [10,11]. The peptides from scorpion venom are classified into disulfide-bridged peptides (DBP) and non-disulfide-bridged peptides (NDBP) [10]. However, the toxins are the most abundant (73%), and those of the *Buthidae* family are the most toxic to humans [12,13]. They are all categorized under DBP and classified into four groups according to the target ion channel in the excitable cells (Na^+^, K^+^, Cl^−^, and Ca^2+^), with specificity and potency varying from one toxin family to another [14,15]. The toxins that mostly contribute to severe scorpionism are the toxins acting on the Na^+^ channels. After their binding, they cause prolonged membrane depolarization and neuronal excitement, leading to stimulation of postganglionic receptors at the level of the sympathetic and parasympathetic nervous systems. Subsequently, the massive liberation of substances, such as acetylcholine, epinephrine, and norepinephrine, disrupts almost all the organisms [16,17]. Furthermore, the scorpion envenomation induces complex clinical signs affecting different organs and systems, classified into three classes according to Suliman et al. [18]. Convulsion, bradycardia, cardiovascular collapse, hypotension, dyspnea, cyanosis, ataxia, and paralysis are among the additional human symptoms observed in patients [18]. The systemic symptoms can be developed within minutes or delayed for as long as 24 h. They are also responsible for major tissular and metabolic complications, such as alveolar destruction, degeneration of the myofibrils, sinusoidal dilation, glomerular disorganization, and high levels of blood sugar and urea [19,20,21,22,23].

Moroccan healthcare workers are currently facing challenges with the management of scorpion envenomations, due to their severity and high incidence [24]. The outcome of treatment of envenomed patients largely depends on the quality and the speed of care. Considering the severity of scorpionism and the capability of toxins to diffuse rapidly, the recommended treatment for scorpion envenoming is antivenom immunotherapy, made up of immunoglobulins or immunoglobulin fragments obtained from the plasma of hyperimmunized animals [25]. The aim is to slow down the spread of the venom, neutralize the toxins, and treat the observed clinical disorders. Despite several reports on the therapeutic benefit of immunotherapy in the treatment of scorpion envenomation [26,27,28,29,30,31,32,33,34], its effectiveness remains controversial in Morocco. The ability of the antivenom to remove venom from the plasma compartment, in less than an hour, was demonstrated [26,35]. Therefore, the healing duration was rapid (less than 4 h) in all patients receiving the antivenom [26]. Hence, the antivenom acts at the level of the various targets of venom by complexing its toxins and induces a redistribution from the tissues to the vascular compartment. The complex is rapidly eliminated via the reticulo-endoplasmic or renal system [36]. Despite the level of evidence in favor of (Fab’)^2^ immunotherapy against scorpion venom [31], it has become imperative to prove the efficacy of the immunotherapeutic approach for the treatment of scorpion envenomations, especially in North Africa.

This study was aimed at defining the toxic potential of the *Am*, *Aah*, and *Bo* dangerous scorpion species in North Africa. It also aimed at the determination of the neutralizing capacity of the selective antivenom used for treatment of envenomation by these species and evaluation of the specificity and efficacy of immunotherapy to neutralize the tissue alterations, induced by the sublethal doses of the scorpion venoms at the level of the main organs of mice: brain, heart, lungs, liver, and kidneys.

## 2. Results

### 2.1. Median Lethal Dose (LD_50_) and Sublethal Doses (sLD) of the Scorpions’ Venoms

The median lethal doses (LD_50_) of *Am*, *Aah*, and *Bo* scorpion venoms, determined by the intraperitoneal route, were found as 302, 597, and 890.1 µg/kg body weight, respectively, showing that the *Am* venom has the highest toxicity, followed by the *Aah* and then the *Bo* venom. The increase in these results is linked to a rise in the sublethal dose values for each venom (135, 255, and 415 µg/kg body weight for *Am*, *Aah*, and *Bo* venoms, respectively) (Table 1).

### 2.2. The Neutralization Capacity of the Scorpion Antivenom (ED_50_)

The scorpionic antivenom revealed high potency to neutralize the lethal effect of 3LD_50_ of each venom injected (Table 2). The antivenom used was more effective against the *Bo* venom (38.73 µg), followed by the *Aah* (57.57 µg) and then the *Am* venom (59.14 µg). Our results consistently demonstrated that the amount of venom (LD_50_ number) neutralized by 1 mL of antivenom was higher with the *Bo* venom compared to the *Aah* and *Am* venoms (77.4, 52.1, and 50.7 LD_50_/mL of antivenom/20 g of mice, respectively).

### 2.3. Clinical Symptoms Observed by the Effect of Scorpion Venoms with or without Antivenom

After the IP injection of the sublethal dose of *Am*, *Aah*, and *Bo* venoms, mice showed mainly neurological symptoms of scorpion envenomation, characterized by paralysis and chills of the lower limbs, hunchback, and agitation at different intensities. When the complex venom and antivenom were injected, the animals showed no signs of envenomation. However, when the antivenom was administered 2 h following the inoculation of venom and installation of the alterations, the symptoms observed were initially similar to those noticed in the first experiment, although the effect of the antivenom ameliorated the majority of these signs, with only mild agitation by the effect of the three venoms, in addition to mild hunchback in the *Am* group (Table 3).

### 2.4. Efficacity of Antivenom at Neutralizing the Physiopathological Effects of Scorpion Venoms

#### 2.4.1. The Tissular Alterations Observed 4 h Following the Experimental Scorpionic Envenomation

**Figure 1:**
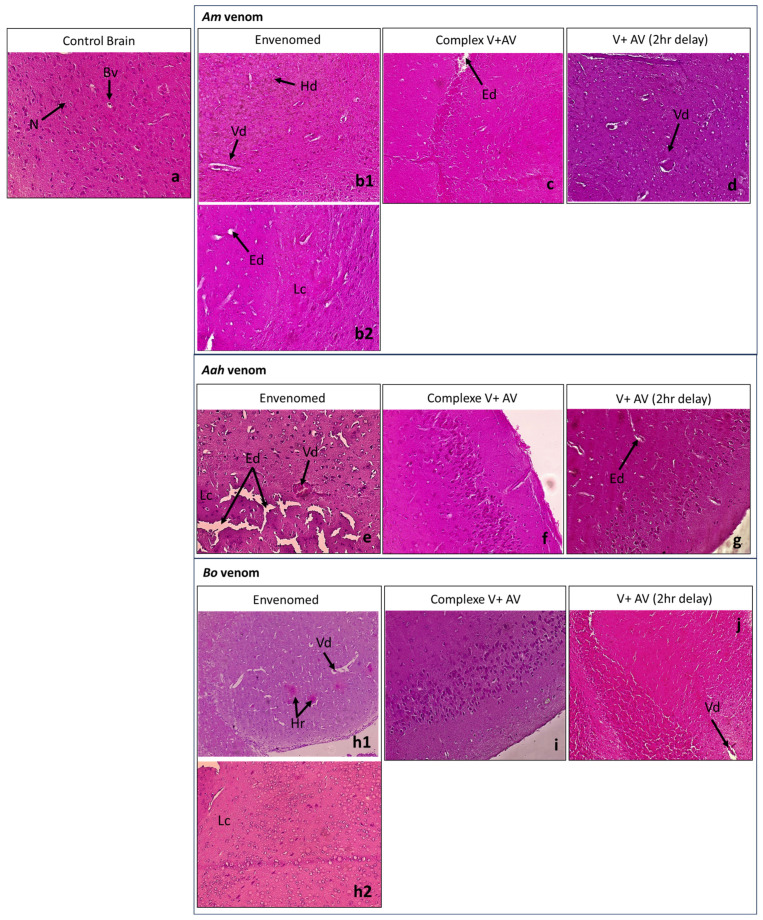
Histological observation of the brains of mice treated with the scorpion venoms (**b1**,**b2**,**e**,**h1**,**h2**), the complex V + AV (**c**,**f**,**i**), and the AV 2 h following the envenomation (**d**,**g**,**j**) (×10). (**a**): Image corresponds to the control group. (**b1**,**b2**,**h1**,**h2**): Images captured from different regions within the same histological section of the group treated with *Am* and *Bo* venoms, respectively. Bv: blood vessel; N: neuron; Hd: hemosiderin deposits; Vd: vasodilatation; Ed: edema; Lc: loss of cellularity; Hr: hemorrhage.

**Figure 2:**
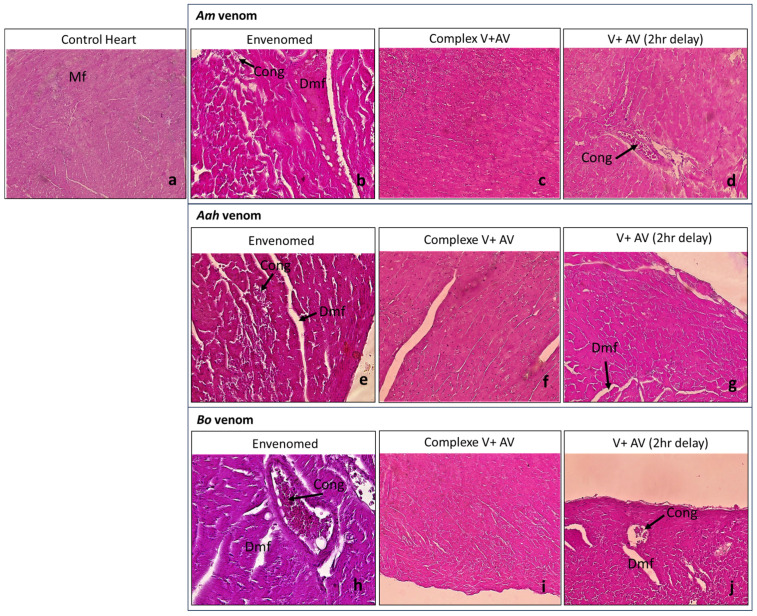
Histological observation of the hearts of mice treated with the scorpion venoms (**b**,**e**,**h**), the complex V + AV (**c**,**f**,**i**), and the AV 2 h following the envenomation (**d**,**g**,**j**) (×40 for the image (**h**) and ×10 for the rest). (**a**): Image corresponds to the control group. Mf: nuscle fiber; Dmf: degeneration of myocardium; Cong: congestion.

**Figure 3:**
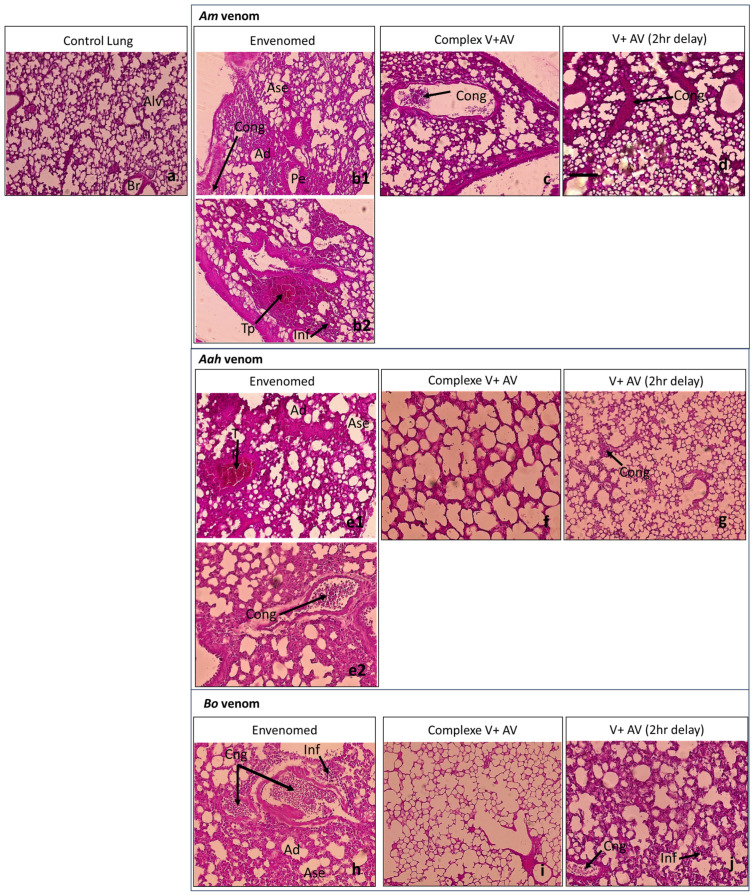
Histological observation of the lungs of mice treated with the scorpion venoms (**b1**,**b2**,**e1**,**e2**,**h**), the complex V + AV (**c**,**f**,**i**), and the AV 2 h following the envenomation (**d**,**g**,**j**) (×40 for all observations except the image (**f**), with ×100). (**a**): Image corresponds to the control group. (**b1**,**b2**,**e1**,**e2)**: Images captured from different regions within the same histological section of the group treated with *Am* and *Aah* venoms, respectively. Alv: alveolar; Br: bronchi; Ad: alveolar destruction; Ase: expansion of alveolar space; Pe: pulmonary emphysema; Cong: congestion; Tp: pulmonary thrombi; Inf: inflammatory cell infiltration.

**Figure 4:**
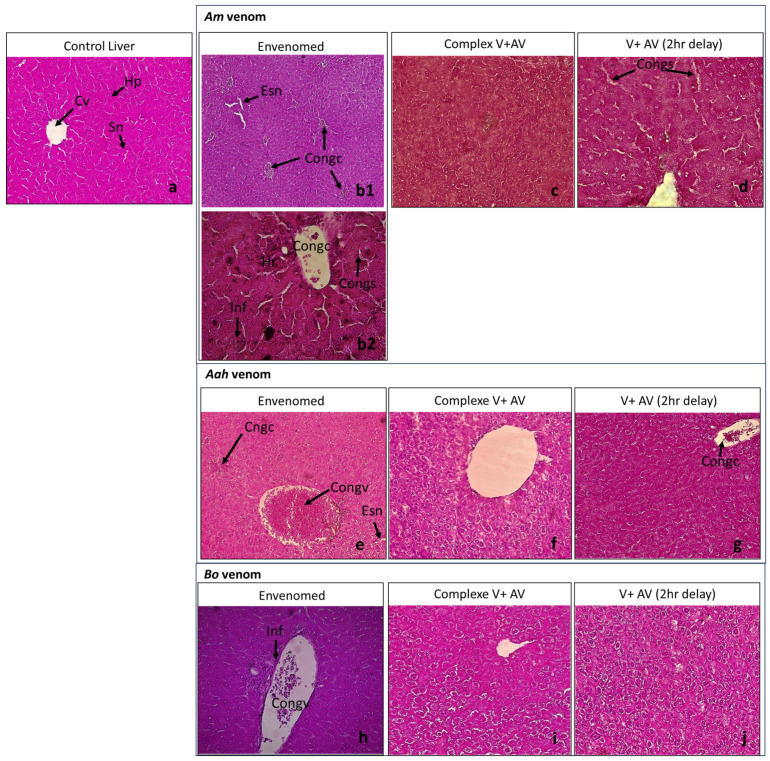
Histological observation of the livers of mice treated with the scorpion venoms (**b1**,**b2**,**e**,**h**), the complex V + AV (**c**,**f,i**), and the AV 2 h following the envenomation (**d**,**g**,**j**) (×10 for the images (**b2**,**d**,**f**,**i**,**j**) and ×40 for the rest). (**a**): Image corresponds to the control group. (**b1**,**b2**): Images captured from different regions within the same histological section of the group treated with *Am* venom. Hp: hepatocyte; Sn: sinusoid; Cv: central lobular vein; Esn: sinusoidal dilation; Congc: centro-lobular congestion; Congs: sinusoidal congestion; Congv: vascular congestion; Inf: inflammatory cell infiltration.

**Figure 5:**
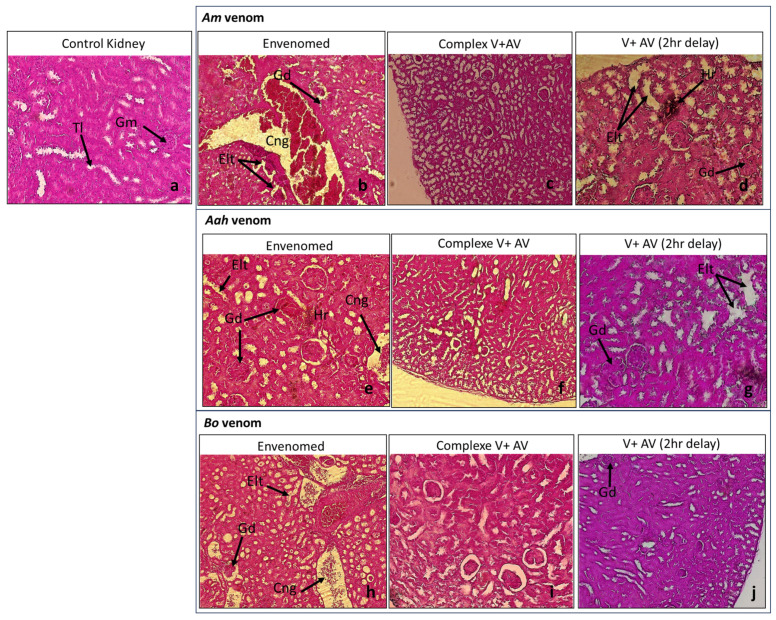
Histological observation of the kidneys of mice treated with the scorpion venoms (**b**,**e**,**h**), the complex V + AV (**c,f,i**), and the AV 2 h following the envenomation (**d**,**g,j**) (×10 for the images (**c**,**f**,**j**), and ×40 for the rest). (**a**): Image corresponds to the control group. Gm: glomeruli; Tl: tubular light; Gd: glomerular destruction; Elt: tubular light widening; Cong: congestion; Hr: hemorrhage.

The injection of the sublethal dose of scorpion venoms showed several alterations with variable intensity compared to the control group (Table 4; Figure 1, Figure 2, Figure 3, Figure 4 and Figure 5). The three venoms caused common damages, which are represented by loss of cellularity with vasodilatation at the level of the brain, degeneration of myofibers and congestion at the level of the heart, alveolar destruction, congestion associated with alveolar space enlargement at the level of the lungs, hepatic necrosis and congestion at the level of the liver, and glomerular disorganization. Congestion and enlargement of the tubular lumen at the level of the kidneys were also detected.

In addition to the common damages mentioned above, there were specific alterations caused by the *Am* venom, such as hemosiderin deposits in the brain, pulmonary emphysema in the lungs, and hepatic hemorrhagic suffusion. However, the *Aah* venom was the only one that caused infiltration of inflammatory cells in the myocardium. The *Bo* venom demonstrated specific alteration of the hemorrhagic areas in the brain.

#### 2.4.2. Neutralizing Ability of the Antivenom Injected in Complexation with the Scorpion Venoms

The administration of the complex venom and antivenom generally revealed an almost normal cellularity in all the organs studied. All histological complications that were observed were not shown by the antivenom against lethality caused by *Am*, *Aah*, and *Bo* scorpion venoms. Although, slight alterations were noticed in the group of mice treated with the *Am* venom incubated with the antivenom, which were a discreet edema in the brain tissue and a less pronounced congestion in the lungs (Table 4; Figure 1, Figure 2, Figure 3, Figure 4 and Figure 5).

#### 2.4.3. Effectiveness of the Antivenom Injected after 2 h Following Scorpion Venom Inoculation

At the level of the brain, the antivenom administered 2 h after the experimental envenomation showed that the previous alterations were partially neutralized with only a local vasodilatation induced by the previous effect of *Am* and *Bo* scorpion venoms. Meanwhile, the edema caused by the *Aah* venom remained, but discreetly (Figure 1). At the level of the heart, the scorpion antivenom was able to considerably neutralize the level of cardiac disorganizations already noted. However, the mice treated with the antivenom still represented degeneration of some myofibers or a less pronounced congestion in groups of *Aah* and *Am* scorpion venoms, respectively. The *Bo* venom group showed a lower level of both lesions (Figure 2). At the level of the lungs, the tissular structure were nearly back to normal due to the effect of the antivenom against the three scorpion venoms, except for the only remaining alteration, which was congestion in the group of mice initially injected with the *Am* and *Aah* venoms. A mild inflammatory cellular infiltration was added in the *Bo* venom group (Figure 3). At the level of the liver, there was a high neutralization of the alteration caused by the *Bo* venom. Microscopically, the hepatic tissue was without damage. This neutralization effect was considered remarkable even in the group treated with the *Am* and *Aah* scorpion venoms, in which only the presence of discreet centro-lobular and sinusoidal congestion was seen, respectively (Figure 4). At the level of the kidneys, the tissular structure remained partially altered with glomerular disorganization due to the effect of the three scorpion venoms, associated with enlargement of the tubular lumen in the *Aah* venom group. However, local hemorrhagic areas were added by injecting the antivenom to mice initially envenomed by the *Am* scorpion venom (Figure 5).

### 2.5. Tissue Localization of the Venom and the Complex Venom–Antivenom

**Figure 6:**
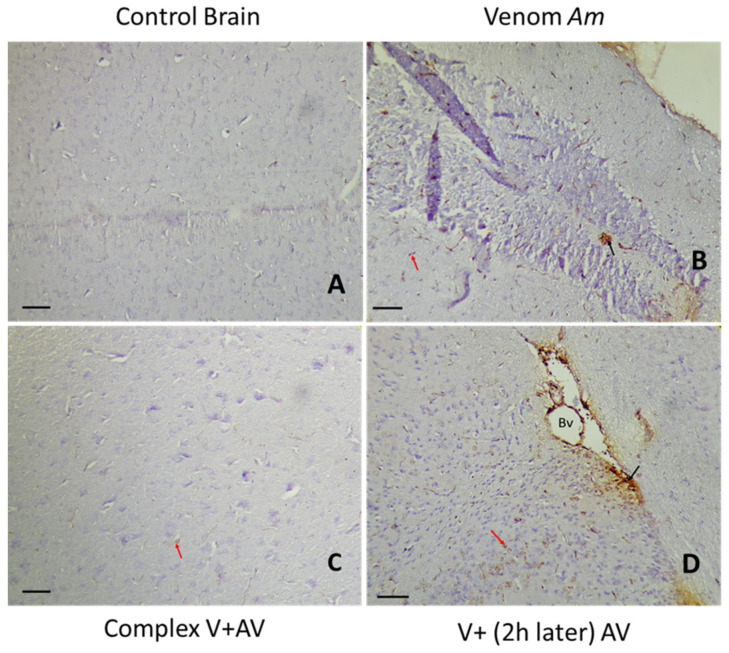
Immunohistochemical detection of *Am* venom (**B**) and the complex venom–antivenom (**C**,**D**) in nerve tissue. (**A**): The control group. The calibration bar = 50 µm. Bv: blood vessel. Brown areas are positive for venom and complex venom–antivenom. Detection in nerve cells (red arrow) and vascular endothelial cells (black arrow).

**Figure 7:**
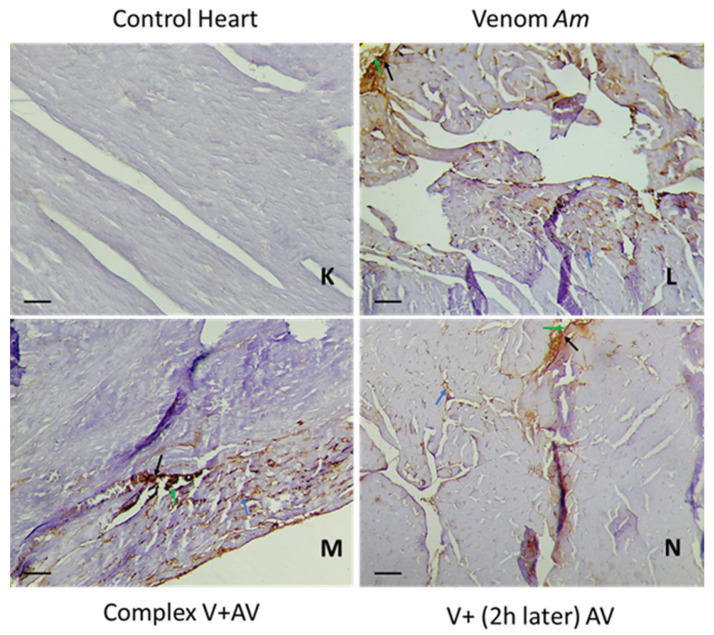
Immunohistochemical detection of *Am* venom (**L**) and the complex venom–antivenom (**M**,**N**) in cardiac tissue. (**K**): The control group. Calibration bar = 50 µm. Brown areas are positive for venom and complex venom–antivenom. Detection in vascular endothelial cells (black arrow), myocardial muscle cells (blue arrow), and inflammatory cells (green arrow).

**Figure 8:**
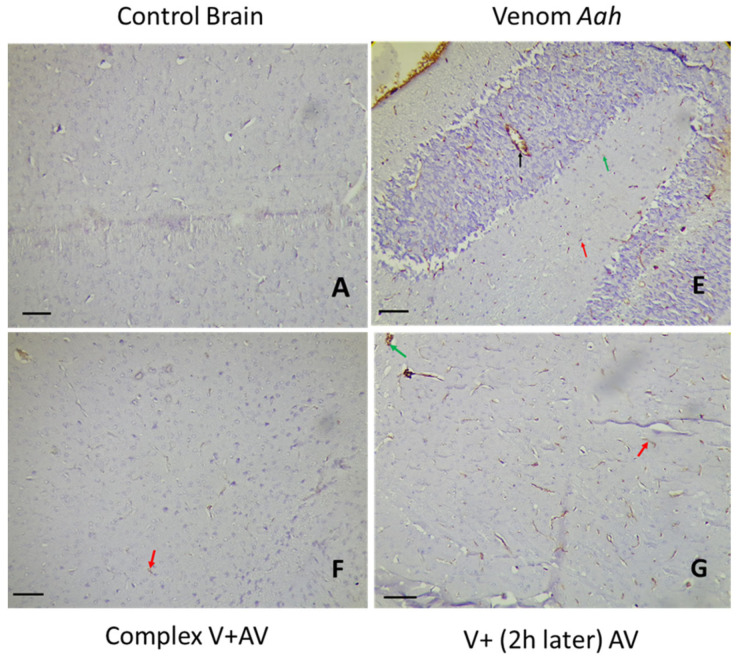
Immunohistochemical detection of *Aah* venom (**E**) and the complex venom–antivenom (**F,G**) in nerve tissue. (**A**): The control group. Calibration bar = 50 µm. Brown areas are positive for venom and complex venom–antivenom. Detection in nerve cells (red arrow), vascular endothelial cells (black arrow), and inflammatory cells (green arrow).

**Figure 9:**
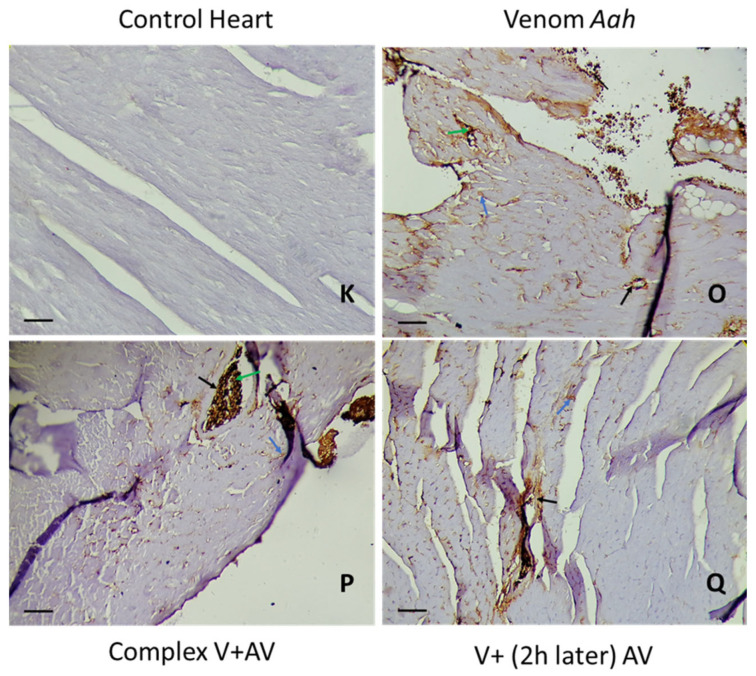
Immunohistochemical detection of *Aah* venom (**O**) and the complex venom–antivenom (**P**,**Q**) cardiac tissue. (**K**): The control group. Calibration bar = 50 µm. Brown areas are positive for venom and complex venom–antivenom. Detection in vascular endothelial cells (black arrow) and myocardial muscle cells (blue arrow), and inflammatory cells (green arrow).

**Figure 10:**
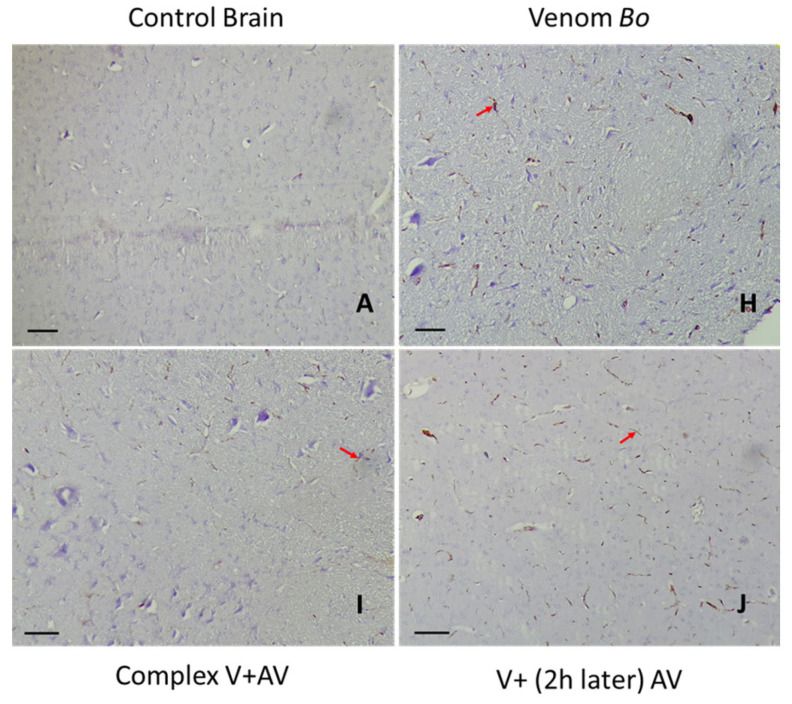
Immunohistochemical detection of *Bo* venom (**H**) and the complex venom–antivenom (**I,J**) in nerve tissue. (**A**): The control group. Calibration bar = 50 µm. Brown areas are positive for venom and complex venom–antivenom. Detection in nerve cells (red arrow).

**Figure 11:**
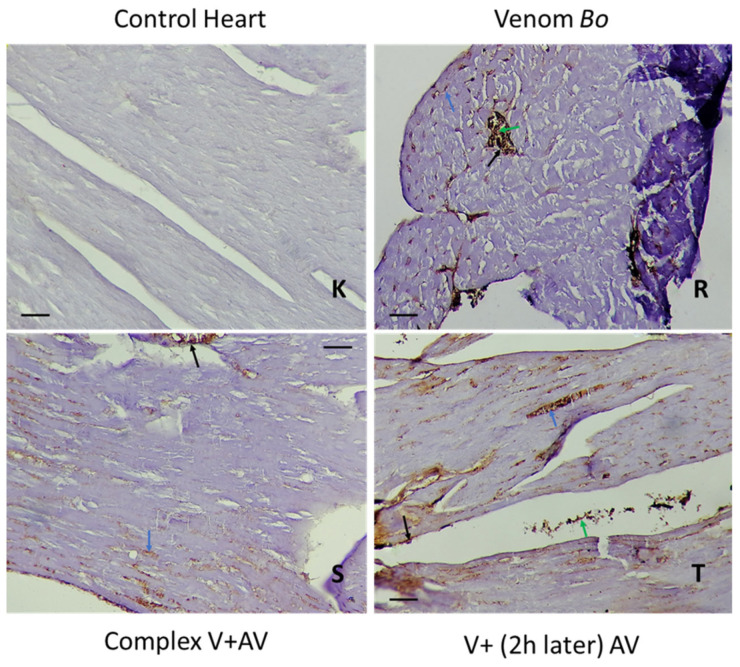
Immunohistochemical detection of *Bo* venom (**R**) and the complex venom–antivenom (**S,T**) in cardiac tissue. (**K**): The control group. Calibration bar = 50 µm. Brown areas are positive for venom and complex venom–antivenom. Detection in vascular endothelial cells (black arrow), myocardial muscle cells (blue arrow), and inflammatory cells (green arrow).

The immunohistochemical study of the brain and heart can not only determine the location of the venom or the complex venom–antivenom at the tissue level, but also indicates the type of cells labeled and the percentage of staining. In correlation with the histopathological changes observed in the same organs, Table 5, Table 6 and Table 7 and Figure 6, Figure 7, Figure 8, Figure 9, Figure 10 and Figure 11 showed the diffusion of staining in endothelial, inflammatory, and nerve/myocardial cells, with significant percentages in the sections of the tissues injected by the sublethal doses of *Am*, *Aah*, or *Bo* venoms. Our results illustrated the presence of a significant amount of venom in the cardiac compared to the nerve tissue. No staining was observed in the sections of the control group. In contrast, the lowest levels of staining were detected in the tissues injected with the combined venom and antivenom. This demonstrates the specificity of the antibody–antigen reaction performed and indicates that it is possible to capture and neutralize scorpion venom. It should be noted that the groups with a medium percentage of staining represent the neutralizing effect of the antivenom by the fixation target (venom) already reached at the tissue level. This may be the result of the time elapsed since the experimental envenomation and the antivenom injection. The results obtained by immunohistochemistry correlated well with the previous histological study revealing the efficacity of the scorpionic antivenom to neutralize tissue alterations already installed in the brain and heart tissues.

#### 2.5.1. Immunohistochemical Staining of the *Am* Venom and the Complex Venom–Antivenom in the Brain and Heart

Immunohistochemical sections of the brain revealed cytoplasmic and membranous staining of *Am* venom and the complex venom–antivenom in nerve and endothelial cells (Table 5). For the group of mice injected with antivenom after 2 h, the presence of the complex was noticed with a lower percentage compared to the group treated with the venom alone, which shows the neutralizing power of the antivenom at the tissue level, toward the *Am* venom. When the complex venom and antivenom was administered to the mouse, a much lower percentage was detected (10%), suggesting that neutralization had already occurred during the incubation of the antivenom and its target (venom).

Following the microscopic observation of cardiac tissue, the staining was related to myocardial, endothelial, and inflammatory muscle cells, with cytoplasmic and membrane localization. A 60% reduction in the staining percentage was observed when *Am* venom was injected alone, compared to the group treated 2 h later with the antivenom (60% vs. 40%). The staining seen in the complex venom–antivenom group showed the specificity of the antibody–antigen reaction, which was already performed (Figure 6 and Figure 7).

#### 2.5.2. Immunohistochemical Staining of *Aah* Venom and Venom and the Complex Venom–Antivenom in the Brain and Heart

The nerve and cardiac tissues were detected by positive staining for *Aah* venom (50% and 80%, respectively) at the levels of nerve/myocardial, inflammatory, and endothelial cells. Staining percentages of 30% and 50% were observed, respectively, in the sections of the brain and heart treated with the antivenom two hours later. The complex already incubated before the injection showed a very small percentage, which was less than 5% at the level of nervous tissue and 30% at the level of the heart (Table 6; Figure 8 and Figure 9).

#### 2.5.3. Immunohistochemical Staining of *Bo* Venom and Venom and the Complex Venom–Antivenom in the Brain and Heart

The immunohistochemistry assay revealed the presence of *Bo* venom and the complex venom–antivenom in nerve/myocardial, inflammatory, and endothelial cells (Table 7). Low staining percentages (10% and 30%, respectively) were observed in the organs (brain and heart) of the groups injected with the complex antigen–antibody already formed before injection. Compared with venom-treated tissues alone, a level of neutralization was presented by the lower percentages obtained in cardiac and nerve tissues (20% and 50%, respectively) following the effect of the antivenom administered after two hours (Figure 10 and Figure 11).

## 3. Discussion

Scorpionism is a major public health problem worldwide, especially in the tropic and subtropic areas. North Africa is home to diverse and dangerous scorpion species—*Androctonus mauretanicus*, *Androctonus australis hector,* and *Buthus occitanus*. Considering the severity of scorpion stings and the controversies surrounding the use of immunotherapy, the present study focused on the evaluation of the toxicity of *Am*, *Aah*, and *Bo* scorpion venoms and the neutralization potency of the antivenom against the histological lesions induced by sublethal doses of the scorpion venoms in the main organs of mice: brain, heart, lungs, liver, and kidneys. The second part of the study was focused on immunohistochemical evaluation of immunotherapy, in relation to the pathophysiological effects induced by the dangerous scorpions in North Africa. 

The toxicological characterization of the three venoms shows that the *Am* venom has a high toxic potency, followed by the *Aah*, then the *Bo* (302 µg/kg, 597 µg/kg, and 890.1 µg/kg body weight, respectively). The *Tityus stigmurus* scorpion, known to be a predominant specie with a highly toxic venom in northeastern Brazil, revealed a similar LD_50_ as *Bo* scorpion venom (LD_50_ = 773 µg/kg) [37,38]. The toxicity of one of Turkey’s dangerous scorpions, *Buthacus macrocentrus,* seems to be less than that of the species studied in North Africa, with a LD_50_ equal to 1250 µg/kg [39]. Despite the characterization of the venoms of *Am*, *Aah*, and *Bo* in previous studies, it was deemed necessary to repeat the tests of toxicity. This is because scorpion venoms are known for their intraspecific variability in terms of toxicity across various parameters. Therefore, we aimed to obtain the specific values of LD_50_ and sLD for these venoms, as they were injected into mice in the following experiments in this study. 

By analyzing the clinical profile observed in the groups of mice, the antivenom was able to reduce the symptoms noted when venoms alone were injected, which were mainly neurological and classified into three categories of scorpion envenomation symptomatology [18]. However, hunchback and chills of the lower limbs were among the first class of signs, while agitation and paralysis of the lower limbs fell under the second and the third classes, respectively. Severe symptoms were absent because of the antivenom injected, even after 2 h of the venom inoculation. However, a low level of agitation was observed in the mice that previously received the *Am*, *Aah*, and *Bo* scorpion venoms, with mild hunchback only in the group that received the *Am* venom. It is important to note that no symptoms were noticed in mice injected with the complex venom and antivenom for the groups of the three scorpion venoms. The study of Revelo et al. confirmed the positive effect of immunotherapy by simultaneous injection of the scorpion venom and antivenom, by eliminating the signs observed previously when the venom of *T. serrulatus* was injected alone. The signs observed previously were not seen, which were irritability, perspiration, piloerection, and respiratory difficulty. In the same study, the capacity of the immunotherapy to reduce the signs observed one hour before the administration of the antivenom was evaluated, in which the animals with respiratory difficulty recovered rapidly [40].

At the level of the histological structure, the neurological, cardiac, pulmonary, hepatic, and renal disorganizations were intense in mice injected with *Am* venom. However, the administration of the complex venom and antivenom prevented the occurrence of almost all histological alterations, whereas the antivenom inoculated for 2 h completely or partially neutralized the tissular manifestations noticed previously. The study of Sami-Merah et al. [41] confirms our results by evaluating the neutralizing effect of antibody fragments, represented by the absence of myocardial and pulmonary damages (hemorrhage, edema, and leukocyte infiltration), after injection of the antivenom 30 min following the inoculation of the whole venom, its toxic fraction FtoxG-50, or the isolated toxin of *Aah* (by using the intraperitoneal route in rats as an animal model). However, alveolar dilation, hypertrophy of myocardial fibers, and alveolar space enlargement persisted. Even in rabbits, when the antivenom was administered one hour after the *Mesobuthus eupeus* scorpion venom, the tissular structure was generally improved, with slight alterations: myocytolysis and edema at the pulmonary and cardiac levels, respectively, with hemorrhagic foci in both organs studied. However, the simultaneous administration of the scorpion antivenom prevented severe alveolar edema, pulmonary thrombi formation, inflammation, and congestion of pulmonary parenchyma, as well as severe myocytolysis, edema, and hemorrhage in the hearts of all treated animals [36].

The evaluation of severe histopathological changes induced by *Am* and *Bo* scorpion venoms was carried out at the nervous, cardiac, pulmonary, hepatic, and renal levels [21]. Most of them were neutralized, although renal parenchyma still showed a disorganization due to its vascularized nature. Bessalem et al. [42] showed that immunotherapy appears to be partially ineffective in the renal cortex, unlike the liver and heart of mice receiving antivenom (consisting of F(ab)_2_ fragments) in a relatively short time (30 min). The neutralizing ability of antivenom in this study is also considered less effective in the lung [43]. The study of Revelo et al. [40] highlighted the maximum concentrations of *T. serrulatus* scorpion venom in the lungs, heart, and spleen—30 min—compared to the kidneys and liver (15 min). However, when the venom was injected alone, its concentration decreased rapidly after 2 h in the serum and other organs studied, until the levels of venom were no longer detectable after 8 h [40], which explains the appearance of various tissue alterations with severe intensity in our study when the antivenom was administered after the distribution of venom at the tissular level.

The different components of the venom are involved in the lesions noted, including toxins that act in the membranes of neuronal and muscle cells and are responsible for cardiac and pulmonary dysfunctions by modifying the action potential of these cells and inducing massive release of neurotransmitters, or by direct action on cardiomyocytes [44]. The cardio-pulmonary response usually involves two phases: the first one is secondary to the release of catecholamines and other vasoconstrictor peptides, leading to hypertension, and the second is represented by structural changes and the morphological and functional performance of myocardial muscles responsible for myocardial failure and a state of shock [45]. The hepatocyte turgescence, on the other hand, is due to increased flow of Ca^2+^ ions that would activate phospholipases [46]. An inflammatory response in the liver and kidneys has been associated with scorpion envenomation syndrome, which is reported by an increase in microvascular permeability and infiltration of inflammatory cells [47]. Phospholipases are considered responsible for membrane hydrolysis [48].

The immunohistochemical analysis of the nerve and cardiac tissue sections can identify the location of the venom in the absence and presence of immunotherapy. The brown staining designating the marking is present in the heart tissue more than nervous tissue. The percentage reflects the presence of marked cells and the level of staining observed under the microscope. The highest percentage was attributed to the cardiac tissue of mice injected with *Aah* venom (80%), while the almost absence of the marking was at the nervous level, with less than 5% following the effect of the complex venom–antivenom injection. It should be noted that the amount of antivenom initially injected into mice was the determined effective dose 50 (which neutralizes a dose of venom referred to as LD_50_), while the amount of venom experimentally administered to mice in complexation with the antivenom or 2 h early was equal to the sublethal dose, which is much lower. As a result, the staining obtained following the administration of the complex reflects the antibody–antigen reaction produced in vivo.

Few studies have determined the locations and targets of the scorpionic venom inoculated during experimental envenomation, although D’suze et al. [49] immunohistochemically detected the scorpion venom *T. discrepans* at the injection site (the inner thigh of rams) in the extracellular matrix, the light of the lymphatic vessels and veinlets. At the liver level, some macrophages migrating to the hepatic sinusoids have been positively marked. This is essentially due to the involvement of severe inflammatory syndrome during scorpionic envenomation by *T. discrepans*.

The antivenom used is a polyvalent preparation containing the F(ab’)_2_ fragments of immunoglobulins produced against the venom of scorpions, including the *Am*, *Aah*, and *Bo* with their different toxic potentials. Severe tissular alterations induced by the venoms have been partially or completely neutralized in all organs studied. However, the efficacy of immunotherapy was proven in our study against the physio-pathological effect of the scorpion venoms in mice via the intraperitoneal route. Various parameters were involved in the effectiveness of antivenom therapy, including the injection route and the natures of the antibody fragments. However, titration tests were conducted to evaluate the neutralizing potency of the scorpion antivenom and determine if sufficient antivenom was administered [50]. The improvement of the specific immunotherapy involved precautions related to the pharmacokinetics of the antivenom, in addition to the conditions of its application. This was intended to improve the accessibility of these antivenoms to reach their targets at the level of different compartments. The efficacity of the IgG immunoglobulin antibodies, F(ab’)_2_ fragments (obtained from pepsin digestion), or Fab fragments (from papain digestion) was different. Although, a pharmacokinetic study of scorpionic antivenom claims that F(ab’)_2_ fragments are most effective due to their rapid extravasation, wide distribution in extracellular space, and prolonged average residence time (MRT). The latter refers to an estimate of the average time that a fabo-therapeutic molecule remains in the body; for F(ab’)_2_, it is about 10.4 (9.1–12.9) days, a value similar to the serum half-life of IgG (estimated at 23 days). This may involve the interaction of the Fc chain with FcRn receptors at the tissue level. The long protection provided by the F(ab’)_2_ fragments is related to other mechanisms since they do not have the Fc chain [51].

The toxicokinetic and toxicodynamic studies of *Aah* scorpion venom showed that in the presence of the antivenom, intraperitoneal injection of either Fab’_2_ or Fab fragments had a similar effect in preventing late symptoms, even after late administration [52]. Although, in terms of neutralizing the venom and redistributing its components from the extravascular compartment to the blood, intravenous injection of Fab’_2_ fragments is much more effective than Fab fragments, and vice versa for the intramuscular pathway [35,52]. Vazquez and al. reported that the intramuscular route (compared to the intravenous route) is not recommended for antivenom administration because the peak of the fragment F(ab)_2_ in the plasma occurred with a remarkable delay [43]. In addition to other evidence, this study confirmed, in its entirety, the neutralization capacity of the antivenom against the severe physio-pathological effects of the most dangerous scorpion species, which can lead to improvement in the design of immunotherapy approaches in North Africa. It has thus become clear that there is no replacement for early specific immunotherapy against the scorpion sting, especially in Morocco.

## 4. Conclusions

In light of our results, the effectiveness of the antivenom to completely or partially neutralize the histological changes was determined against the *Am*, *Aah*, and *Bo* scorpion venoms at the level of the main organs. Therefore, we showed that the venom can be located in nerve/myocardial, endothelial, and inflammatory cells, with high percentages in sections of the nerve and cardiac tissues treated only with venom compared to groups of mice treated with the complex venom–antivenom or the antivenom 2 h early. This study shows promising results for antivenom against the most dangerous scorpion species in North Africa: *Androctonus mauretanicus*, *Androctonus australis hector,* and *Buthus occitanus*. Immunotherapy can represent a specific and recommended treatment, as it has been shown to have neutralizing and curative properties against the scorpionic stings in North Africa.

## 5. Materials and Methods

### 5.1. Venom and Antivenom

The scorpions *Androctonus mauretanicus* (*Am*) and *Buthus occitanus* (*Bo*) were collected from high-risk areas of Morocco and kept, with access to food and water, in the scorpionarium of the Pasteur Institute of Morocco (IPM), located in Tit Mellil. The venoms obtained by the electrical stimulation method [53] were centrifuged at 14,000 r/min for 20 min at 4 °C, lyophilized, and then stored at a temperature of −20 °C. The venom of the Tunisian scorpion *Androctonus australis hector* (*Aah*) was kindly provided in the lyophilized form by the ATheris Laboratories. The F(ab’)_2_ scorpion polyvalent antivenom was generously provided by Silanes Laboratories S.A. in Mexico City, Mexico, where it was manufactured. It is produced in horses through immunization with increasing doses of a mixture of venoms from scorpions implicated in the most severe envenomations in North Africa, including *Androctonus mauretanicus*, *Androctonus australis australis*, *Androctonus garzoni*, *Buthus occitanus*, *Buthus mardochei*, *Buthus occitanus occitanus*, and *Leiurus quinquestriatus*. The lyophilized antivenom was prepared by dissolving the content of one ampoule in 5 mL of 9% NaCl saline solution, then maintained at 4 °C until its use.

### 5.2. Mice

Male Swiss mice (18–22 g) were obtained from the animal unit of the Pasteur Institute of Morocco and kept at an ambient temperature with free access to food and water until used. All animal experiments were in compliance with the guidelines determined by the World Health Organization [54,55] and the local ethics committee at the IPM (agreement number: 8.3. A-2015).

### 5.3. Median Lethal Dose (LD_50_) and Sublethal Doses (sLD) of the Scorpion Venoms

The median lethal dose (LD_50_) was assessed in accordance with the recommendations of the World Health Organization [54] by intraperitoneal (IP) injections of increasing doses of each venom into groups of six mice. The control mice received a saline solution (NaCl 0.9%). The mortality rate was recorded 24 h after envenomation. The LD_50_ was determined by using the software package GraphPad Prism 7 according to the algorithm provided. While respecting the four-parameter logistical equation, the non-linear curve was traced, and constraints were set on minimum and maximum values (0% and 100% mortality). The sublethal dose (sLD) is a maximum concentration of venom inducing the pathophysiological effects without mortality. To define it, decreasing amounts of each venom were injected to groups of mice [56]. All the injections were performed via the intraperitoneal route, and the total volume injected was equal to 500 µL. The results were expressed in µg of venom per kg of body weight.

### 5.4. Median Effective Dose 50% (ED_50_) of the Scorpion Antivenom

The median effective dose 50% of the antivenom was assessed by incubating a fixed dose (3LD_50_) of the *Am*, *Aah*, and *Bo* scorpion venoms with various dilutions of scorpion antivenom for 30 min at 37 °C. A final volume of 500 µL was injected into six groups of mice via the intraperitoneal route. After 48 h, the survival ratio was recorded and analyzed by GraphPad Prism 7 software using the non-linear regression analysis with a variable-slope dose–response curve. The ED_50_ was expressed as the volume (µL) of antivenom protecting 50% of mice against known lethal amounts of scorpion venom (3LD_50_) or as the LD_50_ amount of venom completely neutralized by 1 mL of antivenom.

### 5.5. Experimental Design

For each venom, all experiments were performed in groups of 4 mice. In the first one, the mice were injected with the sublethal dose of the scorpion venom. In the second group, the sLD of the venom was incubated (at 37 °C for 30 min) with the ED_50_ that neutralized 1 LD_50_ per mouse. In the third group, the effective dose (ED_50_) was administered to mice 2 h following the injection of venom (sLD). The clinical signs were observed throughout the manipulation. All animals were treated by intraperitoneal injection of a total volume equal to 500 µL and anesthetized 4 h after the last experimental act. The control group received a physiological saline solution (NaCl 0.9%).

### 5.6. Histopathological Studies

The main organs of mice: brain, heart, lungs, liver, and kidney, were carefully harvested, fixed in 10% formaldehyde solution (for 24 h), dehydrated through graded ethanol series, and then impregnated with paraffin. The sections, with a thickness of 4 μm, were stained using hematoxylin-eosin and observed under a photonic microscope for histological examination [57].

### 5.7. Immunohistochemistry

The paraffin-embedded tissue sections of the brain and heart were deparaffinized and rehydrated through xylol and alcohol series (5 min each). Sections were rinsed twice with phosphate-buffered saline (PBS) for 5 min, then proceeded to antigen retrieval by using Tris buffer solution (pH 6.0) and incubating at 95 °C for 10 min. The sections were allowed to cool for 20 min. The blocking of the endogenous peroxidase was assessed by keeping the tissues in 3% hydrogen peroxide (H_2_O_2_) for 15 min. The non-specific protein activity was blocked with the use of bovine serum albumin 5% (BSA) for 5 min. Then, the sections were incubated at 37 °C (for 10 min) with the scorpionic antivenom for the sections of tissues taken from groups of mice injected only by the scorpion venom. Secondary antibodies were then added and incubated in a humidified chamber (the sections of organs obtained from mice injected with the scorpion venom and antivenom in complex or separately were treated directly by the second antibody). As a chromogen, diaminobenzidine (DAB) was used to revel the antigen/antibody reaction. The sections were rinsed at the end of each step with two changes of phosphate-buffered saline (PBS; 5 min), except after the protein blocking [58,59]. The visualization and discussion of the results were performed by using a light microscope at ×40 magnification.

## Figures and Tables

**Table 1:** Determination of intraperitoneal LD_50_ and sLD of *Am*, *Aah*, and *Bo* scorpion venoms (with 95% confidence intervals).

	*Am* Venom	*Aah* Venom	*Bo* Venom
LD_50_ (µg/kg)	302 ± 60	597 ± 41	890.1 ± 10.7
sLD (µg/kg)	135 ± 1.9	255 ± 6.1	415 ± 87

The results are expressed as means ± SD.

**Table 2:** Neutralization capacity of the antivenom used against *Am*, *Aah*, and *Bo* scorpion venoms.

ED_50_	*Am* Venom	*Aah* Venom	*Bo* Venom
in (µL) *	59.14 ± 1.28	57.57 ± 0.29	38.73 ± 0.55
in (number of LD_50_ per mL of antivenom) **	50.7	52.1	77.4

The ED_50_ (median effective dose) is expressed in µL of antivenom protecting 50% of mice *, and in number of LD_50_ of venoms completely neutralized by 1 mL of antivenom **.

**Table 3:** Neurological signs observed after injection of *Am*, *Aah*, and *Bo* venoms with or without scorpionic antivenom.

		Paralysis of the Lower Limbs	Chills of the Lower Limbs	Hunchback	Agitation
*Am*	*Am* Venom	+++	++	++	+++
	Complex Venom + Antivenom	-	-	-	-
	Venom + Antivenom (2 h delay)	-	-	+	+
*Aah*	*Aah* Venom	++	+	+++	+++
	Complex Venom + Antivenom	-	-	-	-
	Venom + Antivenom (2 h delay)	-	-	-	+
*Bo*	*Bo* Venom	++	+	++	+++
	Complex Venom + Antivenom	-	-	-	-
	Venom + Antivenom (2 h delay)	-	-	-	+

Note. The following symbols represent the severity of signs: ‘’-‘’ signifies absence, ‘’+’’ indicates mild severity, ‘’++’’ denotes moderate severity, while ‘’+++’’ signals severe severity for the signs.

**Table 4:** Histopathological effects of the scorpion venoms and the efficacity of the antivenom injected in complexation or after 2 h of experimental envenomation.

		*Am* Scorpion Venom			*Aah* Scorpion Venom			*Bo* Scorpion Venom		
	Tissular alterations	*Am* Venom	Complex V + AV	V+ AV (2 h Delay)	*Aah* Venom	Complex V + AV	V+ AV (2 h Delay)	*Bo* Venom	Complex V + AV	V+ AV (2 h Delay)
Brain	Loss of cellularity	x	-	-	x	-	-	x	-	-
	Vasodilatation	x	-	x	x	-	-	x	-	x
	Edema	x	x	-	x	-	x	-	-	-
	Hemosiderin deposits	x	-	-	-	-	-	-	-	-
	Hemorrhage	-	-	-	-	-	-	x	-	-
Heart	Degeneration of myofibers	x	-	-	x	-	x	x	-	x
	Congestion	x	-	x	x	-	-	x	-	x
	Infiltration of inflammatory cells	-	-	-	x	-	-	-	-	-
Lungs	Alveolar destruction	x	-	-	x	-	-	x	-	-
	Alveolar space enlargement	x	-	-	x	-	-	x	-	-
	Infiltration of inflammatory cells	x	-	-	-	-	-	x	-	x
	Congestion	x	x	x	x	-	x	x	-	x
	Pulmonary emphysema	x	-	-	-	-	-	-	-	-
	Pulmonary thrombi	x	-	-	x	-	-	-	-	-
Liver	Centro-lobular congestion	x	-	-	x	-	x	x	-	-
	Sinusoidal dilation	x	-	-	x	-	-	-	-	-
	Sinusoidal congestion	x	-	x	-	-	-	-	-	-
	Infiltration of inflammatory cells	x	-	-	-	-	-	x	-	-
	Necrosis	x	-	-	x	-	-	x	-	-
	Hemorrhage	x	-	-	-	-	-	-	-	-
Kidneys	Enlargement of tubular lumen	x	-	x	x	-	x	x	-	-
	Glomerular disorganization	x	-	x	x	-	x	x	-	x
	Congestion	x	-	-	x	-	-	x	-	-
	Hemorrhage	-	-	x	x	-	-	-	-	-

Note. The following symbols represent the histological effects: ‘’-‘’ indicates absence, and ‘’x’’ signifies presence.

**Table 5:** Immunohistochemical profile of the brain and heart of mice treated with the *Am* venom and antivenom.

	Group	Type of Staining	Type of Cell	Percentage
Brain	*Am* Venom	Cytoplasmic Membrane	− Nerve − Endothelial	40
	Complex Venom + Antivenom	Cytoplasmic Membrane	− Nerve	10
	Venom + Antivenom (2 h delay)	Cytoplasmic Membrane	− Nerve − Endothelial	30
Heart	*Am* Venom	Cytoplasmic Membrane	− Myocardial − Endothelial − Inflammatory	60
	Complex Venom + Antivenom	Cytoplasmic Membrane	− Myocardial − Endothelial − Inflammatory	20
	Venom + Antivenom (2 h delay)	Cytoplasmic Membrane	− Myocardial − Endothelial − Inflammatory	40

**Table 6:** Immunohistochemical profile of the brain and heart of mice treated with the *Aah* venom and antivenom.

	Group	Type of Staining	Type of Cell	Percentage
Brain	*Aah* Venom	Membrane	− Endothelial − Inflammatory − Nerve	50
	Complex Venom + Antivenom	Membrane	− Nerve	Less than 5
	Venom + Antivenom (2 h delay)	Membrane	− Inflammatory − Nerve	30
Heart	*Aah* Venom	Cytoplasmic Membrane	− Endothelial − Inflammatory − Myocardial	80
	Complex Venom + Antivenom	Cytoplasmic Membrane	− Endothelial − Inflammatory − Myocardial	30
	Venom + Antivenom (2 h delay)	Cytoplasmic Membrane	− Endothelial − Myocardial	50

**Table 7:** Immunohistochemical profile of the brain and heart of mice treated with the *Bo* venom and antivenom.

	Group	Type of Staining	Type of Cell	Percentage
Brain	*Bo* Venom	Cytoplasmic Membrane	− Nerve	40
	Complex Venom + Antivenom	Cytoplasmic Membrane	− Nerve	10
	Venom + Antivenom (2 h delay)	Cytoplasmic Membrane	− Nerve	20
Heart	*Bo* Venom	Cytoplasmic Membrane	− Endothelial − Inflammatory − Myocardial	60
	Complex Venom + Antivenom	Cytoplasmic Membrane	− Endothelial − Myocardial	30
	Venom + Antivenom (2 h delay)	Cytoplasmic Membrane	− Endothelial − Inflammatory − Myocardial	50

## Data Availability

Data is contained within the article.
